# Diaphragmatic Hernia as a Prenatal Feature of Glycosylphosphatidylinositol Biosynthesis Defects and the Overlap With Fryns Syndrome – Literature Review

**DOI:** 10.3389/fgene.2021.674722

**Published:** 2021-06-07

**Authors:** Przemyslaw Kosinski, Milena Greczan, Aleksandra Jezela-Stanek

**Affiliations:** ^1^1st Department of Obstetrics and Gynecology, Medical University of Warsaw, Warsaw, Poland; ^2^Department of Pediatrics, Nutrition, and Metabolic Diseases, Children’s Memorial Health Institute, Warsaw, Poland; ^3^Department of Genetics and Clinical Immunology, National Institute of Tuberculosis and Lung Disease, Warsaw, Poland

**Keywords:** glycosylphosphatidylinositol biosynthesis defects, GPIBDs, congenital diaphragmatic hernia, CDH, Fryns syndrome

## Abstract

Fryns syndrome is an autosomal recessive multiple congenital anomaly syndrome, with diaphragmatic defects and secondary lung hypoplasia as cardinal features. Despite it was reported first in 1979, its exact etiology has not been established to date. With this review, we would like to draw attention to the prenatal presentation of multiple congenital anomalies syndromes, resulting from defects in the synthesis of glycosylphosphatidylinositol anchors, to be considered in a prenatal assessment of fetuses with DH and Fryns-like phenotype.

## Introduction

Fryns syndrome (FRNS, %229850) is an autosomal recessive multiple congenital anomaly syndrome. It was reported first in 1979 in two siblings with numerous congenital anomaly, including coarse facies, diaphragmatic hernia, absence of lung lobulation, and distal limb deformities (Fryns and Van den Berghe, 1979). To date, the exact etiology of FRNS (FS) has not been established, and genetic heterogeneity remains highly probable. From the clinical perspective, diaphragmatic defects and secondary lung hypoplasia are cardinal features (Fryns, 1987).

Congenital diaphragmatic hernia (CDH) is a developmental discontinuity of the diaphragm. As a result, the abdominal viscera may herniate into the chest and leads to lung hypoplasia. It occurs in approximately 1 in 3,000 live births but results in high morbidity and mortality, in neonatal and infancy period (Wynn et al., 2014). The most common localization is the posterolateral left side of the diaphragm (75–90% of cases), but the defect can also be right-sided (10–15% of cases) or even bilateral (1–2% of cases) (Deprest et al., 2011). The pulmonary vasculature malformation are the main cause of CDH-related pulmonary hypertension (Sluiter et al., 2012). Moreover, increased vascular resistance and decreased surface area for gas exchange are observed (Hislop and Reid, 1976).

Unfortunately, the pathogenesis of CDH has not been established definitively, and encompass wide range of genetic disorders, from chromosomal defects, noted in at least 10–30% cases, including polyploidies, trisomy 18, partial trisomy 5, partial trisomy 20 or tetrasomy 12p (Pallister-Killian syndrome) (Graham and Devine, 2005; Pober, 2008; Kosinski and Wielgos, 2017) to monogenic syndromes (as Apert, CHARGE, Coffin-Siris, Goltz, Swyer, Brachmann-Cornelia De Lange, Simpson-Golabi-Behmel, Donnai-Barrow, Mathew-Wood, Jarcho-Levin, and Fraser). The influence of genetics in CDH was recently reviewed by Yu et al. (2020). This comprehensive review explains the genetic contributions to CDH are highly heterogeneous and suggests CDH genes are often transcription factors, genes involved in cell migration or the components of extracellular matrix. In cases of multiple abnormalities, malformations may occur in all major organ systems (Pober et al., 2005; Pober, 2007; Taylor et al., 2009). Since the etiology of CDH varies and is difficult to establish, with this review, we would like to draw attention to the prenatal presentation of multiple congenital anomalies syndromes, resulting from defects in the synthesis of glycosylphosphatidylinositol (GPI) anchors, as a possible cause, and to be considered in a prenatal assessment of fetuses with DH and Fryns-like phenotype.

## Diaphragmatic Hernia as a Manifestation of Fryns Syndrome

In 1987 following criteria were suggested by Fryns (1987) to establish the diagnosis of FRNS: polyhdramnios, often occurring in the presence of normal fetal growth, in an infant with characteristic facial dysmorphism – a coarse face, a broad flat nasal bridge (but a large nose anteriorly), a short upper lip, a small jaw, a cleft lip and palate, and poorly shaped auricles with attached earlobes. The phalanges are distally hypoplastic with rudimentary and dysplastic nails. The cardinal features within internal organs are diaphragmatic defects with secondary lung hypoplasia. Moreover, malrotation of the intestine, duodenal or multiple atresias and a bicornuate uterus may be noted. Cloudy corneas, facial hirsutism and pulmonary segmentation defects all help in establishing the diagnosis. The eye findings in the syndrome were reviewed by Pierson et al. (2004), who found corneal clouding in nine out of 15 patients, anophthalmia in two and retinal dysplasia in two. Subtle skeletal abnormalities are also part of the condition (Kershisnik et al., 1991; Tsukahara et al., 1995).

Fryns (1995) points out that growth parameters may be above the 75th centile at birth with apparent macrocephaly. The author suggested that the finding of diaphragmatic hernia with intrauterine growth retardation is against the diagnosis (Fryns, 1995). Vargas et al. (2000) described discordant phenotype for diaphragmatic hernia in monozygotic twins. A few cases have survived the neonatal period and are severely retarded (Bamforth et al., 1989; Cunniff et al., 1990; Hanssen et al., 1992; Van Hove et al., 1995). Dingens and Fryns (1999) reported that the girl first described by Hanssen et al. (1992) had died at the age of 10 years in status epilepticus. She was found to have hematometra.

The individual presented by Davis and Samarakkody (2002) (with no facial photographs available), was diagnosed with Fryns syndrome based on constellation of physical anomalies, as coarse facial feature, fifth digits’ hypoplasia and nail absence, tricuspid regurgitation, diaphragmatic defects, as well as Hirschsprung’s disease. The finding of a diaphragmatic defect and renal abnormalities might alert the astute investigator to the possibility of this syndrome, but note that Siebert et al. found that 13–27% of infants with a diaphragmatic defect had a urinary tract anomaly (Siebert et al., 1990). Willems et al. (1991) reported a possible case with the diaphragm reduced to a fibrous web without an overt hernia. In some cases CDH might be the only ultrasound abnormality detected prenatally.

In a review of 23 patients with a diaphragmatic hernia (Congenital Diaphragmatic Hernia Study Group, 2002), seven patients underwent surgical repair at an average age of 7.5 days (range, 6 h to 14 days); the mortality rate was as high as 83% in patients with both CDH and FS compared to 33% in patients with unilateral isolated CDH (Neville et al., 2002).

McPherson et al. reported a case of Pallister-Killian syndrome (resulted from mosaic isochromosome 12p) with some Fryns syndrome features (facial dysmorphism and digital anomalies) and pointed out the possibility of diagnostic challenge (McPherson et al., 1993). Stratton et al. (1994) and Rodriguez et al. (1994) noted similar cases with Pallister–Killian syndrome and re-emphasized the latter point.

Three cases have had osteochondrodysplasia (mostly delayed ossification) (Kershisnik et al., 1991; Slavotinek et al., 2005). Wilgenbus et al. (1994) reported two fetuses with features of the condition where there were no diaphragmatic hernias, but one of them had severe lung hypoplasia. Bartsch et al. (1995) reported similar two more cases with Fryns syndrome but without lateral diaphragmatic defects. Records of 1,833 liveborn patients with CDH from 83 hospitals revealed Fryns syndrome in 23 cases witch constitutes for 1.3% (Neville et al., 2002). On the other hand, CDH among Fryns syndrome patients is present in approximately 76% to 89% of reported cases (Cunniff et al., 1990; Van Hove et al., 1995).

While Ramsing et al. (2000) delineated three fetuses between 15 and 31 weeks of gestation with FS features (Cases 3–5). They also reported a family where two fetuses had a more severe form or a different condition (Cases 1 and 2). Several cases have been reported with features of the discussed condition and chromosome aberrations, including duplication of 1q24–31 (Clark and Fenner-Gonzales, 1989), terminal 6q deletion (Krassikoff and Sekhon, 1990), a submicroscopic deletion of 1q41–42 (Kantarci et al., 2006) and partial trisomy 22 (Dean et al., 1991). It should be noted that mosaic trisomy 9 also shares these features. Ladonne et al. (1996) reported a case of trisomy 22 with features of Fryns syndrome. de Beaufort et al. (2000) presented a patient with some features of the condition who had a partial trisomy 22. There is an excellent review by Slavotinek describing diagnostic features of Fryns syndrome (Slavotinek, 2004).

Based on previously published articles, Lin et al. (2005) suggested a definition of the Fryns phenotype, encompassing the combination of six criteria: diaphragmatic defects, pulmonary hypoplasia, recognizable facial features, distal digital hypoplasia, associated anomalies (polyhydramnios, cloudy corneas, cleft lip/palate and genitourinary, cardiovascular and cerebral malformations), and affected sibs. The presence of four among mentioned characteristics may provide a strict clinical definition of FS, while three constitute a broad definition of FS. To date, no gene had been clearly associated with FS. Thus the diagnosis typically remains clinical after the exclusion of chromosomal aberrations using classical karyotyping and/or array comparative genomic hybridization (aCGH) (Jezela-Stanek et al., 2020).

## Fryns Syndrome Phenotype in Glycosylphosphatidylinositol Biosynthesis Defects (GPIBDs)

Recently, several patients with syndromic CDH, harboring pathogenic variants in *PIGN* (Phosphatidyl Inositol Glycan Anchor Biosynthesis, Class N), *PIGA* (Phosphatidyl Inositol Glycan Anchor Biosynthesis, Class A), and *PIGV* (Phosphatidyl Inositol Glycan Anchor Biosynthesis, Class V) gene have been characterized (Longoni et al., 1993; Slavotinek, 1993; Brady et al., 2014; McInerney-Leo et al., 2016; Thompson and Cole, 2016; Reynolds et al., 2017; Alessandri et al., 2018; Witters et al., 2018; Bayat et al., 2020). These molecular diagnosed have expanded the genetic spectrum of Fryns-like phenotype to be part of congenital disorder of glycosylations (CDG), which encompass GPI-anchor biosynthesis defects (GPIBD).

Glycosylphosphatidylinositol is a phosphoglyceride that anchors proteins to the outer leaflet of the cellular membrane. GPIBDs form a group of heterogeneous diseases with a common underlying cause of inappropriate GPI-anchors biosynthesis or modification. Correct GPI biosynthesis and modification determine GPI-anchored proteins’ expression on the cell surface and thus determines the proper antigen presentation, cell adhesion, and signal transduction (Alessandri et al., 2018). According to the clinical and laboratory findings, GPIBDs have been historically divided into two main subgroups: hyperphosphatasia with mental retardation syndrome (HPMRS) – a group of GPIBDs with elevated serum alkaline phosphatase activity (ALP) (mutations in *PIGV, PGAP2, PGAP3, PIGO, PIGW*, and *PIGY*) and multiple congenital anomalies hypotonia seizures (MCAHS) – with normal serum ALP (mutations in *PIGA*, *PIGN*, *PIGT).* However, the number of GPIBSDs that cannot be divided into these two groups is growing (Knaus et al., 2018). Accordingly, the terminology is questioned and no longer recommended.

PIGN-CDG and PIGA-CDG are MCAHS, characterized by severe developmental delay, early-onset intractable seizures, global hypotonia, congenital cardiac, gastrointestinal, renal and central nervous system (CNS) anomalies, nystagmus, failure to thrive and facial dysmorphism. In some individuals, brachytelephalangy with nail hypoplasia, joint contractures and CDH have also been described (Alessandri et al., 2018; Knaus et al., 2018). Surprisingly, ALP levels are elevated in some individuals with PIGA-CDG and borderline in some individuals with PIGN-CDG (Knaus et al., 2018).

PIGV-CDG is HPMRS (also known as Mabry syndrome), characterized by the constantly elevated ALP, intellectual disability, seizures with facial dysmorphia, brachytelephalangy and nail hypoplasia. In some individuals, Hirschprung disease, renal and anorectal malformations and CDH have been described (Reynolds et al., 2017).

No specific treatment is available so far for these conditions, and early death may occur.

The summary of described cases is presented in Table 1. Please note that the diagnoses cited by us are the original ones described in the discussed publications (that is why the terminology is diverse).

**TABLE 1:** Summary of reported to date cases with Fryns syndrome phenotype due to glycosylphosphatidylinositol biosynthesis defects (GPIBDs).

References: no. of cases, sex	Prenatal clinical features	Postnatal clinical features	Genotype
***PIGN* gene (cytogenetic location: 18q21.33 phenotype: Multiple congenital anomalies-hypotonia-seizures syndrome 1 MIM 614080)**			
Alessandri et al., 2018: 3			
Patient 2, female	Polyhydramnios	Dysmorphic facial features, cloudy corneas and terminal hypoplasia of V digits with fingernails absence; right-sided diaphragmatic hernia (diagnosed by chest X-ray) presented with refractory hypoxemia and died at 24 h of life	Deletion-spanning exons 5–7 of *PIGN*
Patient 5, female	22 WG: left diaphragmatic hernia associated with bilateral pyelectasis and polyhydramnios	Hypoplasia of of hands and feet’s distal phalanges, hirsutism and facial dysmorphism, small, low set and dysplastic ears, small nose with a flat nasal bridge, and macrostomia	Frameshift variant in *PIGN* [NM_176787.4:c.(421dup), p.(Ile141Asnfs*10)]
Patient 6, male (brother of Patient 5)	Left diaphragmatic hernia and polyhydramnios, with dilated and hyperechogenic kidneys	Microcephaly, symmetric growth retardation, hypoplastic distal phalanges and nails (of hands and feet), micropenis, wide and short neck, facial dysmorphic features: anteverted nares, macrostomia, a posterior cleft palate, and dysplastic ears; ventricular septal defect and two bilobed and hypoplastic lungs	As above
McInerney-Leo et al., 2016: 3			
NSGC-7.3, female	15 WG: omphalocele, left-sided CDH 17 WG: right-sided cleft lip, cystic and mildly echogenic kidneys, and pulmonary hypoplasia	Pregnancy was terminated	Stop mutation in exon 21 (NM_176787.4: c.1966C > T: p.Glu656X) and a splice site mutation following exon 18 (NM_176787.4: c.1674 + 1G > C) in the *PIGN* gene
NSGC-7.4, male (brother of NSGC-7.3)	13 WG: left-sided diaphragmatic hernia, omphalocele, kidneys anomalies, pulmonary hypoplasia, and unilateral cleft lip; increased nuchal translucency (3.39 mm, >95th centile for gestation)	Pregnancy was terminated	As above
Individual COLL-2.3, female	13 WG: raised nuchal translucency (8 mm, >95% centile), cystic hygroma, and fetal ascites 15 WG: severe septated cystic hygromata, a small exomphalos, moderately hyperechogenic bowel, echogenic kidneys, femur length on the 5th centile	Autopsy: facial dysmorphism (with hypertelorism), distal phalanges of V fingers; multiple internal organ malformations: brain, lungs and heart, bilateral posterior diaphragmatic herniae	Stop mutation in exon 9 of *PIGN* (NM 176787.4: c.694A > T, p.Lys232X)
Brady et al., 2014: 1			
Male		Pregnancy was terminated at 16 WG due to DH	Homozygous splice site mutation in *PIGN*: (NM_012327.5:exon16: c.1574fl1G > A; NM_176787.4:exon17: c.1574fl1G > A).
***PIGA* gene (cytogenetic location: Xp22.2 phenotype: Multiple congenital anomalies-hypotonia-seizures syndrome 2 MIM 300868)**			
Bayat et al., 2020: 1			
Patient 3, male (his sibling, patients 2, had craniofacial dysmorphic features, brachytelephalangy with nail hypoplasia, but no CDH)		Pregnancy was terminated at 16 weeks of gestation due to a severe CDH	Splice site variant c.-63 + 1G > C in *PIGA*
***PIGV* gene (cytogenetic location: 1p36.11 phenotype: Hyperphosphatasia with mental retardation syndrome 1 MIM 239300)**			
Reynolds et al., 2017: 1			
Sibling 1, female (sibling 2: elevated alkaline phosphatase, providing further evidence for a diagnosis of HPMRS, not Fryns syndrome)	20 WG: left-sided diaphragmatic hernia, head to lung ratio of 0.68, pelviectasis, heart anomaly (not confirmed as a result of DH) 32 WG: polyhydramnios	Coarsening of face, broad nasal base, prominent globes, V-shaped concha, and overfolded ear helix, telecanthus, broad mouth, hypoplastic V finger with absent nails	Compound
			Heterozygous variants within the *PIGV* gene [NM_017837.3]: a
			A maternally inherited missense variant in exon 3 [c.1022 C > A; p.
			Ala341Glu (GCA > GAA)] and a paternally inherited missense variant in
			Exon 4 [c.1253 C > A; p.Ala418Asp (GCT > GAT)]

## Summary

Due to the complex nature and not fully recognized etiology of “Fryns syndrome”, some confusion about the terminology exists. When it is a clinical entity, “Fryns-like phenotype” term should be used. Otherwise, Fryns syndrome is a disorder with presumed autosomal recessive inheritance. It is thus of primary importance to distinguish between a clinical or molecular diagnosis. In all individuals/fetuses within the spectrum of Fryns syndrome phenotype genetic diagnostics is rational.

It has to be underlined that a genetic abnormality must be suspected in each case of CDH diagnosed prenatally, when accompany with other malformations. Except for CDH, the clinical presentation of Fryns-like phenotype includes pulmonary hypoplasia, craniofacial dysmorphism, cleft lip/palate, brachytelephalangy accompany with nail hypoplasia, and various other internal organ malformations. Although no major genetic cause has been identified for FRNS, biallelic pathogenic variants in *PIGN*, *PIGV* and X-linked in *PIGA*, all encoding a component of the GPI-anchor biosynthesis pathway, have been identified in several probands with a phenotype fitting with FRNS.

During the genetic evaluation of fetuses that manifest developmental anomalies, it is mandatory to check for chromosomal aberrations (and perform karyotype and/or chromosomal microarray) and also to consider monogenic disorders (Committee on Genetics and the Society for Maternal-Fetal Medicine, 2016; Hay et al., 2018). All of the GPIBDs presented above, means caused by pathogenic variants in the *PIGN*, *PIGV*, and *PIGA* genes, are clinically very heterogenic and congenital malformations are not among their primary characteristics in many other affected individuals. All these syndromes belong to neurodevelopmental disorders and manifest predominantly with facial dysmorphism, global developmental disorders (especially speech and language), muscular hypotonia and seizures, as well as cerebral or cerebellar atrophy in PIGN-CDG, minor CNS anomalies in PIGA- and PIGV-CDG (Siebert et al., 1990; Knaus et al., 2018; Bayat et al., 2020).

Given the high mortality rate resulting from severe congenital malformations, reliable and early genetic diagnosis is crucial. It only allows for determining the recurrence risk (which is 25% for autosomal recessive disorder and even up to 50% for X-linked disorders, if the genetic status of mothers is unknown) and perform genetic testing in subsequent pregnancies. Thus, we propose to include GPI biosynthesis defects in the differential diagnosis during the prenatal evaluation of fetuses with diaphragmatic defects, especially in families with positive family history. When isolated cases are evaluated, the additional ultrasound features that may direct the diagnosis are large for gestational age, hypertelorism, cleft lip/palate, atrial/ventricular septal defects, urogenital anomalies and hand/feet abnormal positioning. However, we wonder how much safer it would be, keeping in mind the complexity of Fryns syndrome, to utilize the term “Fryns syndrome – like phenotype”.

## Author Contributions

PK: manuscript writing and literature review. MG: manuscript writing. AJ-S: concept of the manuscript, writing, and literature review. All authors contributed to the article and approved the submitted version.

## Conflict of Interest

The authors declare that the research was conducted in the absence of any commercial or financial relationships that could be construed as a potential conflict of interest.
